# Supplementary material for: Small RNA in sperm–Paternal contributions to human embryo development
Source: Nat Commun. 2025 Jul 17;16:6571. doi: 10.1038/s41467-025-62015-2 (PMC12267487; doi:10.1038/s41467-025-62015-2)
Supplement: Supplementary file 1 — Supplementary Information [file 41467_2025_62015_MOESM1_ESM.pdf]

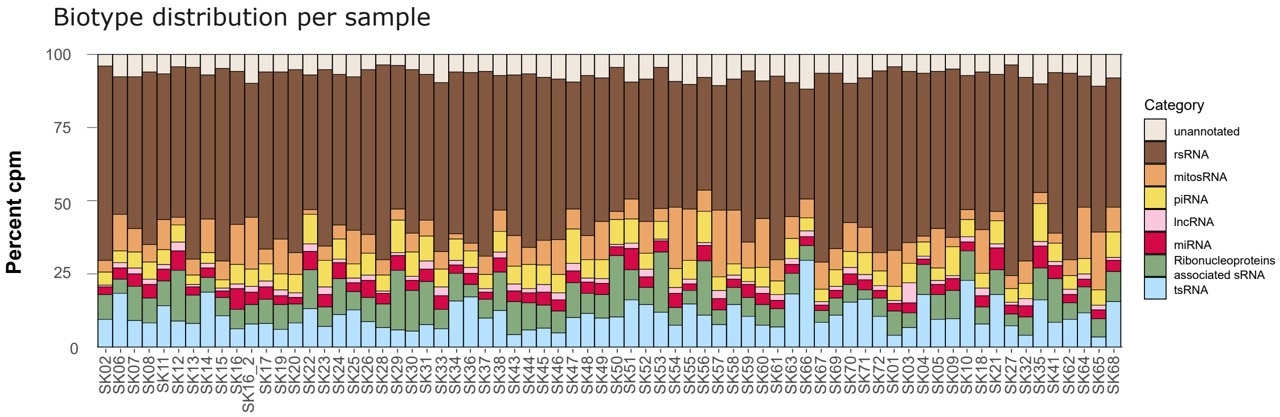

Supplementary Figure 1. **Variation of sperm-borne small RNA profile between individuals.** Stack bar of percent normalised sequence (cpm) per biotype of small RNA annotated as described in methods section, where each value on the x axis represents one biological sample, n=70.

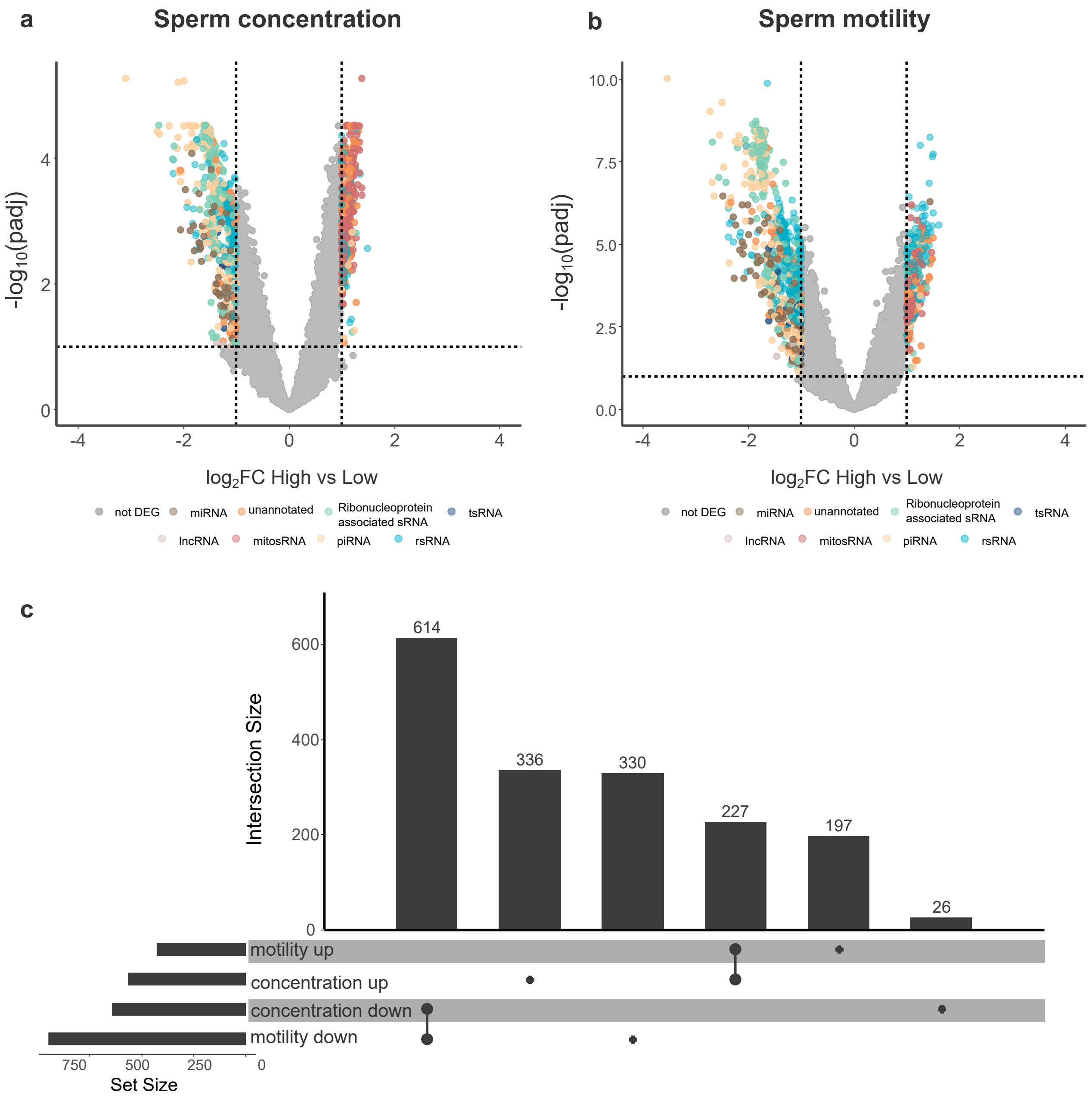

Supplementary Figure 2. **Comparison of differentially expressed sperm sRNA between sperm concentration in ejaculate and number of motile sperm after density gradient. a** Volcano plot showing differentially expressed RNA comparing high sperm concentration to low (>/≤ 16 million sperm/mL), with biotypes colour coded. **b** Volcano plot showing differentially expressed sRNA comparing sperm with high number of motile sperm compared to low, with biotypes colour coded (≥/< 5 million motile sperm). **c** Upset plot showing sequences from significant differentially expressed sRNA and whether they are shared or unique for the four groups; upregulated in sperm motility, upregulated in sperm concentration, downregulated in sperm concentration or lastly downregulated in sperm motility. Upregulated are sequences with an adjusted p-value of <0.1 and a $\log_2$ fold change above 1, whereas downregulated are sequences with an adjusted p-value of <0.1 and a $\log_2$ fold change below -1. DEG=Differentially expressed genes. Differential expression was performed with DESeq2 as described in methods section.

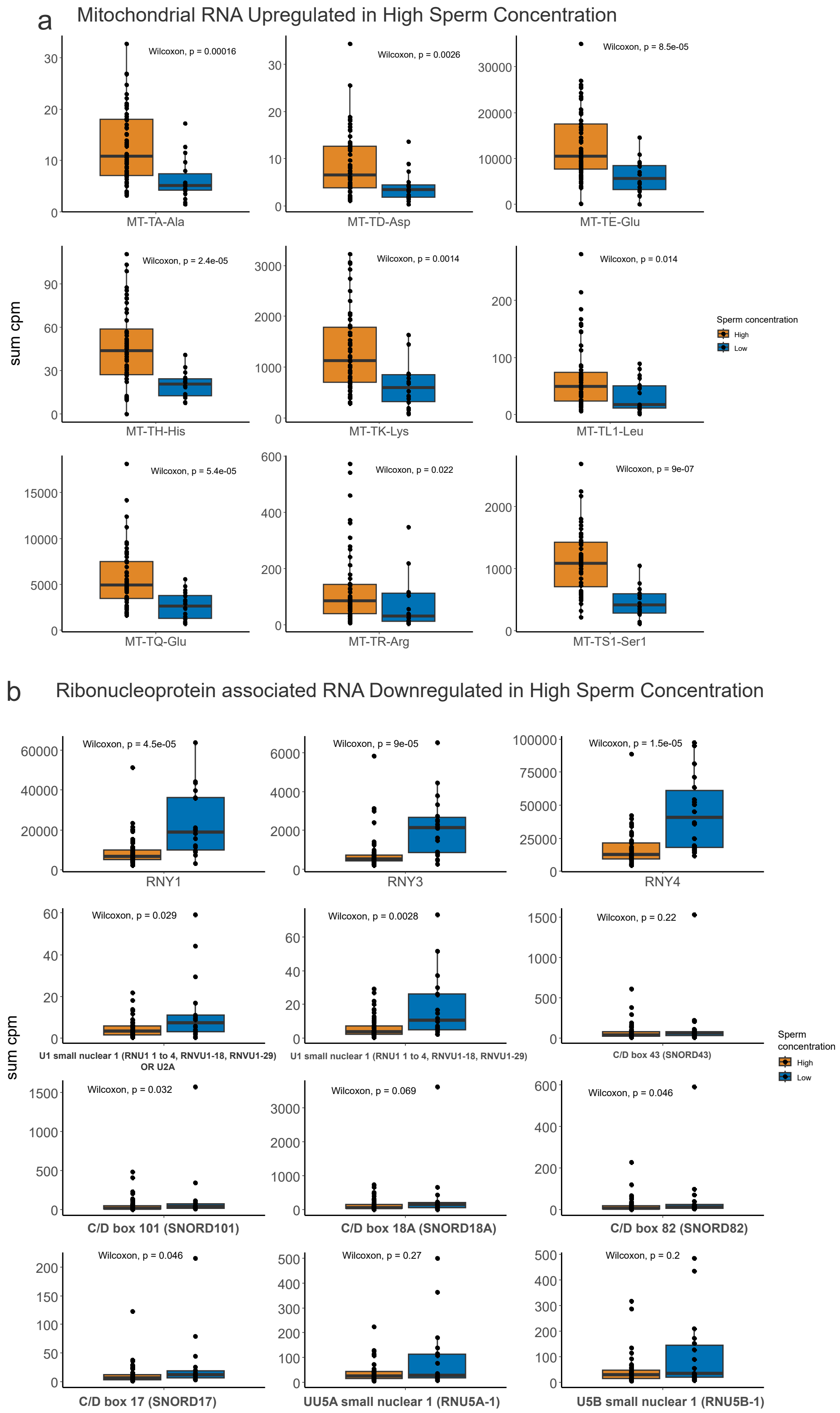

Supplementary Figure 3. **All differentially expressed sRNAs mapping to mitosRNA and ribonucleoprotein associated sRNA respectively**. **a** All differentially upregulated mitosRNA in high sperm concentration (> 16 million sperm/mL. **b** All differentially downregulated ribonucleoprotein associated sRNA in high sperm concentration. Orange=group of high sperm concentration, blue=group of low sperm concentration (≤16 million sperm/mL). Data is presented as sum of normalised sequences (cpm) for each sperm sample by indicated genomic origin. High sperm concentration n=51, low sperm concentration n=18. Line in box plot represents the median, hinges show the first and third quartiles and whiskers extend to the largest value unless values are above 1.5 times the inter-quartile range. P-values shown as Wilcoxon was produced with Wilcoxon nonparametric test (two-sided).

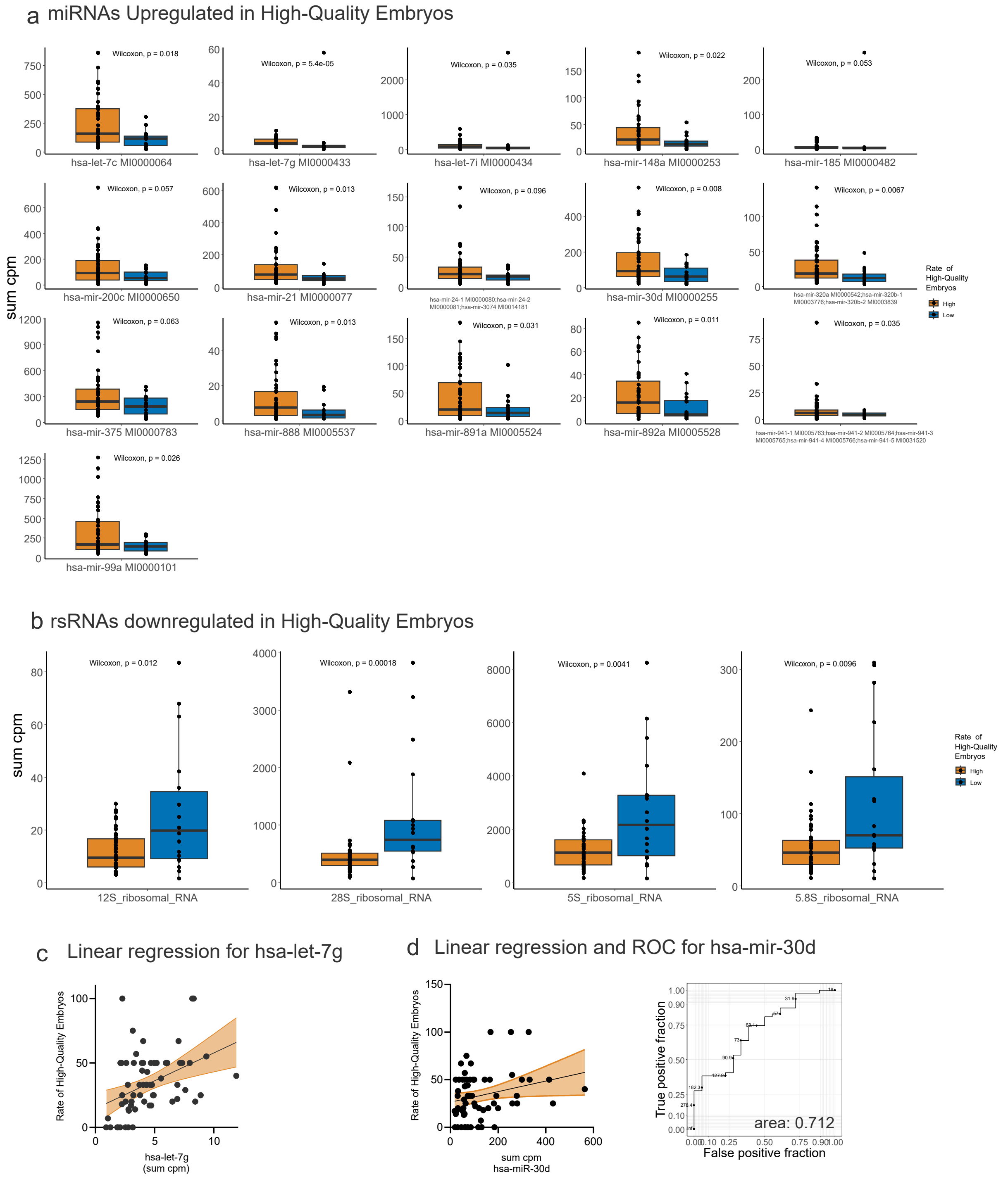

Supplementary Figure 4. **All differentially expressed sRNAs mapping to miRNA and rRNA respectively. a** All miRNAs differentially upregulated in high rate of high-quality embryos (≥ 20%). **b** All rRNA differentially downregulated in high rate of high-quality embryos. **c** Linear regression (R^2=0.16, pvalue=0.0008, y=4.313*x+14.62) for hsa-let-7g without sample SK16, with a cpm twelve times higher than the mean cpm of all other samples. **d** Linear regression (R^2=0.065 and p-value=0.0399) and area under ROC (0.712) for hsa-mir-30d. Orange=group of high rate of high-quality embryos, blue=group of low rate of high-quality embryos (< 20%). Data is presented as sum of normalised sequences (cpm) for each sperm sample by indicated genomic origin. High rate of high-quality embryos n=47, low rate of high-quality embryos n=17. Line in box plot represents the median, hinges show the first and third quartiles and whiskers extend to the largest value unless values are above 1.5 times the interquartile range. In linear regression, coloured area represents 95% confidence interval. P-values shown as Wilcoxon was produced with Wilcoxon nonparametric test (two-sided).

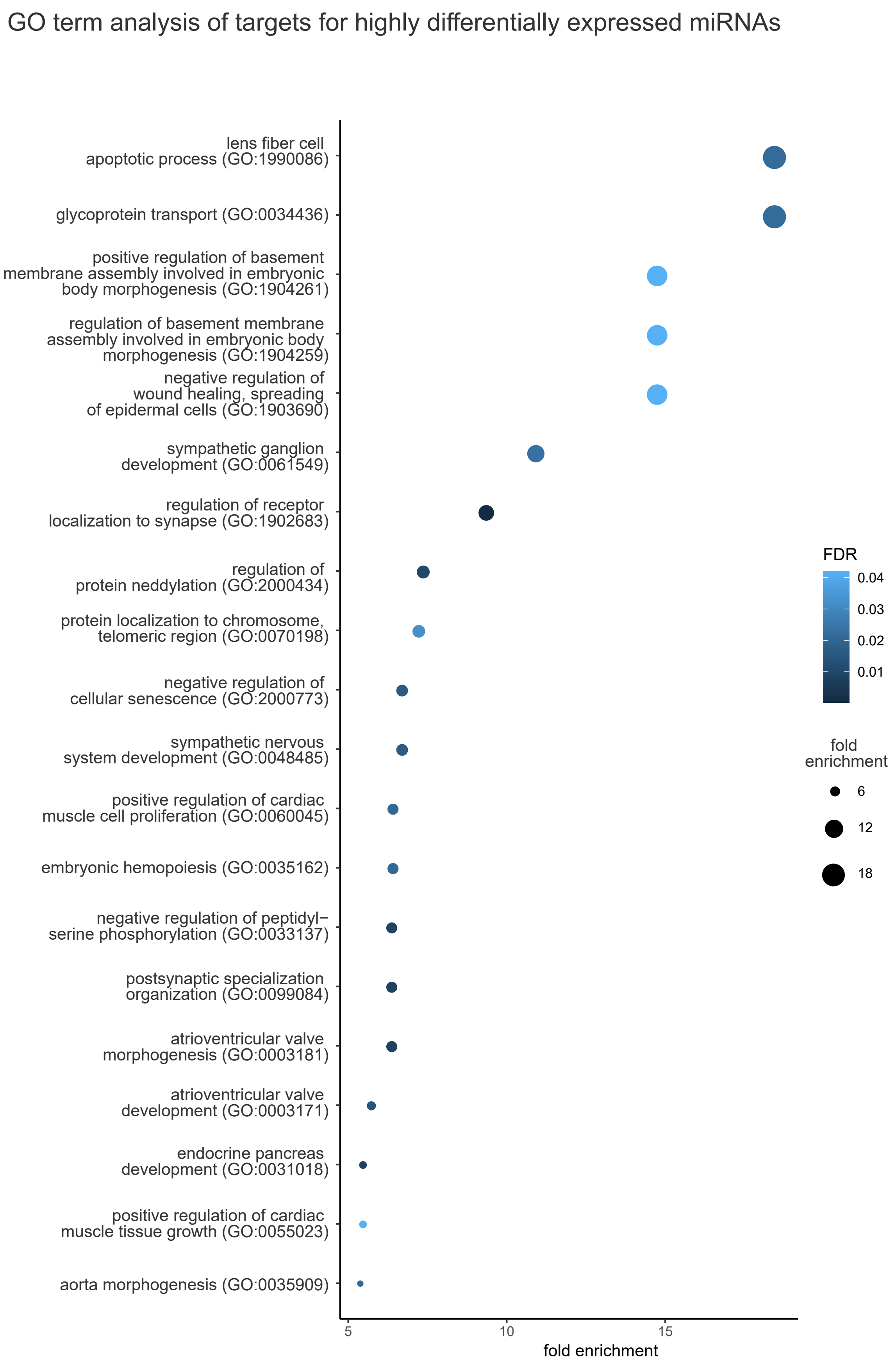

Supplementary Figure 5. **GO term analysis of targets for miRNAs of high significant difference in high versus low rate of high-quality embryos**. MiRNAs are hsa-let-7g, hsa-mir-30d and hsa-mir-320b (Figure 5a). Targets were found with TargetScan 8.0 (Supplementary Table 9) and GO term analysis was performed with PantherDB. GO= Gene Ontology, FDR= False Discovery Rate.

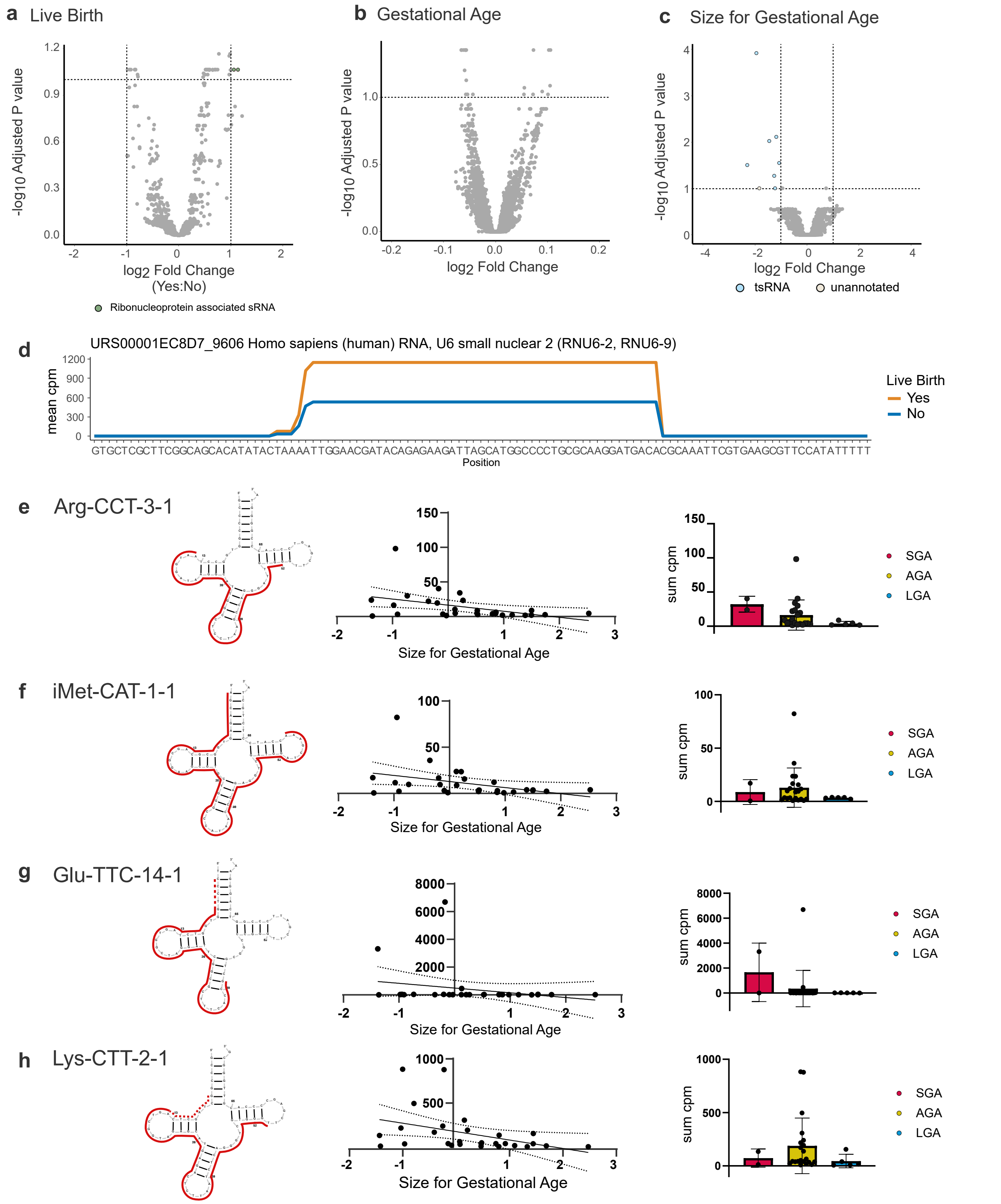

Supplementary Figure 6. **Differentially expressed sequences in live birth, gestational age and size for gestational age. a** Differential expression live birth (yes/no). **b** Differential expression comparing gestational age in days. Log Fold Change is based off each increase in the value of age in days. **c** Differential expression comparing size for gestational age. Log Fold Change is based off each increase in the value of size for gestational age (z-score). **d** Differentially expressed sequences in live birth mapped against snRNA U6. Lines show mean normalised sequences (cpm) in live birth (yes/no). **e** TsRNA sequence mapping to Arg-CCT-3-1 (left), have a linear regression to size for gestational age with R^2=0.1879, p-value=0.0212, y=-8.811*x+16.48 (middle). **f** TsRNA mapping to iMet-CAT-1-1 (left), have a linear regression to size for gestational age with R^2=0.1529, p-value=0.0396, y=-6.579*x+12.82 (middle). **g** Two tsRNA sequences mapping to Glu-TTC-14-1 (left), have a linear regression to size for gestational age with R^2=0.05811, p-value=0.02165, y=-341.3*x+485.6 (middle). **h** Four tsRNA sequences mapping to Lys-CTT-2-1 (left), have a linear regression to size for gestational age with R^2=0.1583, p-value=0.0360, y=-95.23*x +182.0 (middle). Differential expression are sequences with an adjusted p-value of <0.1 and a $\log_2$ fold change above 1 or below -1. Differentially expressed sequences are colour coded to biotype in volcano plots. SGA=small for gestational age, AGA=average for gestational age, LGA=large for gestational age. The red lines represent the position of the identified tsRNA on their corresponding tRNA (left) in (e), (f), (g) and (h), where dashed bars show sequence isoform. Live birth yes n=28, live birth no n=42. In linear regression, dotted area represents 95% confidence interval. Differential expression was performed with DESeq2 as described in the methods section.

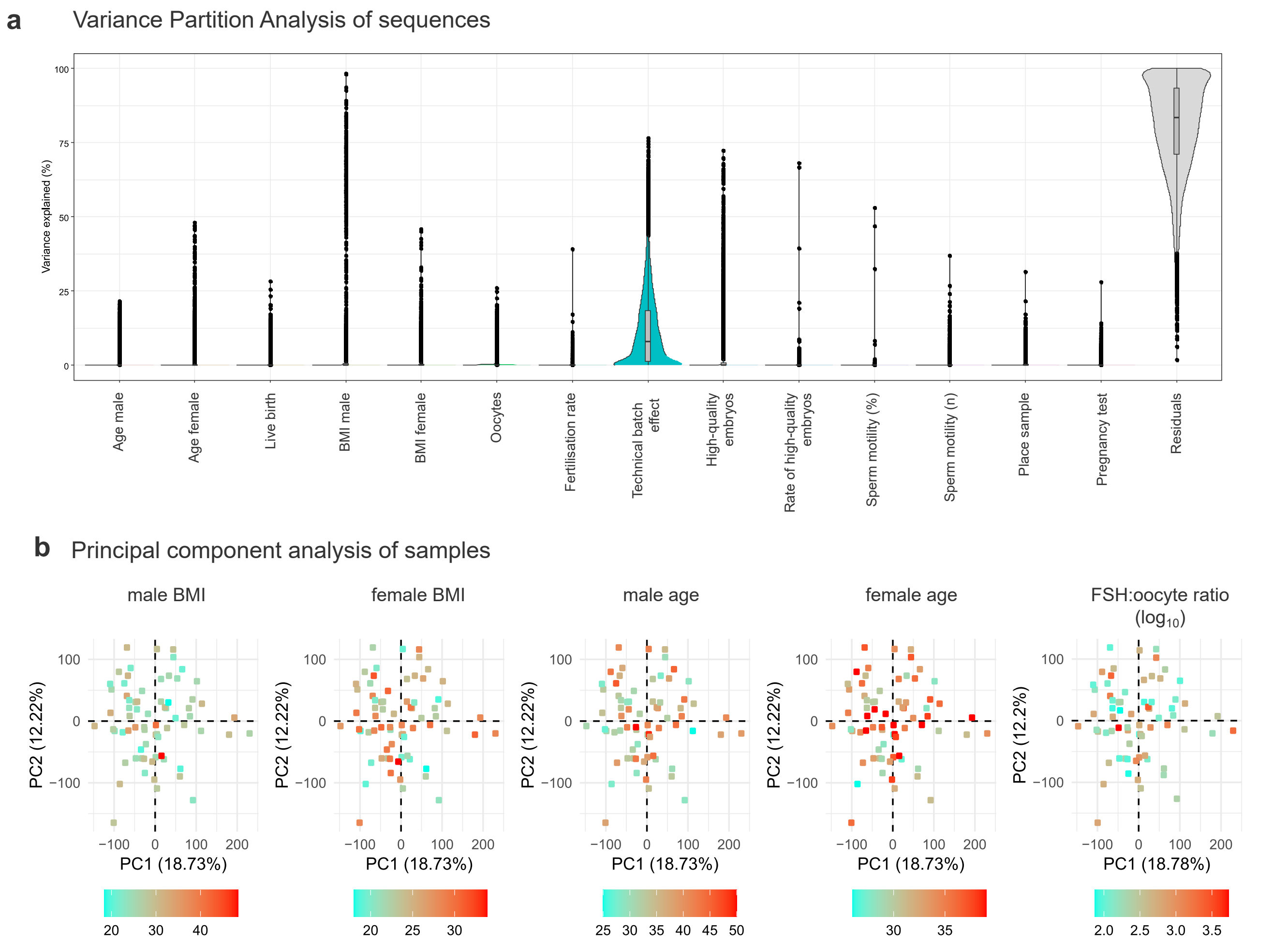

Supplementary Figure 7. **Variables influencing data variation. a** Variance Partition Analysis for sequencing data. Performed in R version 4.3.2 with variancePartition version 1.32.5. Age=years binned in years of five (male) or three (female), live birth=yes/no, BMI=categorised in underweight, normal weight, overweight and obese, oocytes=no., fertilisation rate=≥/<70%, high-quality embryos=no., rate of high-quality embryos=≥/< 20%, Sperm motility (%) = Progressively motile sperm before gradient (≥/< 30%), Sperm motility (n) = Progressively motile sperm after gradient (≥/< 5 million), place sample=home/clinic, pregnancy test=yes/no. **b** PCA (principal component analysis) plot of sRNA sequencing data. Colour represents male BMI (body mass index), female BMI, male age, female age and FSH:oocyte ratio presented in $\log_{10}$ scale. FSH = follicle-stimulating hormone. FSH:oocyte ratio = Total dose hormone (international units) / n oocytes)). All PCAs show principal component 1 on x and principal component 2 on y. n=70. In (a) and PCA plot concerning hormone, the sample with deviating hormonal treatment is excluded.
