# Supplementary material for: Small RNA in sperm–Paternal contributions to human embryo development
Source: Nat Commun. 2025 Jul 17;16:6571. doi: 10.1038/s41467-025-62015-2 (PMC12267487; doi:10.1038/s41467-025-62015-2)
Supplement: Supplementary file 3 — Description of Additional Supplementary Files [file 41467_2025_62015_MOESM3_ESM.pdf]

Description of Additional Supplementary Files

File Name: Supplementary Data 1
Description: sRNA Data from Study

File Name: Supplementary Data 2
Description: Couples divided by High and Low Sperm Concentration

File Name: Supplementary Data 3
Description: DEGs Sperm Concentration High vs Low (>/<16 million sperm/mL)

File Name: Supplementary Data 4
Description: Couples divided by High and Low Fertilisation Rate

File Name: Supplementary Data 5
Description: DEGs Fertilisation Rate High vs Low (>/< 70% embryos per retrieved oocyte)

File Name: Supplementary Data 6
Description: Couples divided by High and Low Rate of High-Quality Embryos

File Name: Supplementary Data 7
Description: DEGs for Rate of High-Quality Embryos (>/<20%)

File Name: Supplementary Data 8
Description: Upregulated miRNA in Rate of High-Quality Embryos (based on Supplementary Data 6)

File Name: Supplementary Data 9
Description: miRNA Targets Predicted using TargetScanHuman Version 8.0

File Name: Supplementary Data 10
Description: miRNA Relevant for Male Fertility

File Name: Supplementary Data 11
Description: DEGs of sperm sRNA based on Live Birth, Gestational Age and Size for Gestational Age

File Name: Supplementary Data 12
Description: Normality of data used for Figure 3a, 3d, 5a, 5d, Sup. Fig. 3, Sup. Fig. 4 a and b
