# Supplementary material for: Small RNA in sperm–Paternal contributions to human embryo development
Source: Nat Commun. 2025 Jul 17;16:6571. doi: 10.1038/s41467-025-62015-2 (PMC12267487; doi:10.1038/s41467-025-62015-2)
Supplement: Supplementary file 5 — Reporting Summary [file 41467_2025_62015_MOESM5_ESM.pdf]

nature portfolio

Corresponding author(s): Professor Anita Öst

Last updated by author(s): Jun 13, 2025

Reporting Summary

Nature Portfolio wishes to improve the reproducibility of the work that we publish. This form provides structure for consistency and transparency in reporting. For further information on Nature Portfolio policies, see our Editorial Policies and the Editorial Policy Checklist.

Statistics

For all statistical analyses, confirm that the following items are present in the figure legend, table legend, main text, or Methods section.

n/a	Confirmed	
☐	☒	The exact sample size (*n*) for each experimental group/condition, given as a discrete number and unit of measurement
☐	☒	A statement on whether measurements were taken from distinct samples or whether the same sample was measured repeatedly
☐	☒	The statistical test(s) used AND whether they are one- or two-sided<br>*Only common tests should be described solely by name; describe more complex techniques in the Methods section.*
☐	☒	A description of all covariates tested
☐	☒	A description of any assumptions or corrections, such as tests of normality and adjustment for multiple comparisons
☐	☒	A full description of the statistical parameters including central tendency (e.g. means) or other basic estimates (e.g. regression coefficient) AND variation (e.g. standard deviation) or associated estimates of uncertainty (e.g. confidence intervals)
☐	☒	For null hypothesis testing, the test statistic (e.g. *F*, *t*, *r*) with confidence intervals, effect sizes, degrees of freedom and *P* value noted<br>*Give P values as exact values whenever suitable.*
☒	☐	For Bayesian analysis, information on the choice of priors and Markov chain Monte Carlo settings
☒	☐	For hierarchical and complex designs, identification of the appropriate level for tests and full reporting of outcomes
☒	☐	Estimates of effect sizes (e.g. Cohen's *d*, Pearson's *r*), indicating how they were calculated

Our web collection on statistics for biologists contains articles on many of the points above.

Software and code

Policy information about availability of computer code

Data collection	FASTQ generation v1.0.0 by Illumina for producing sequencing files
Data analysis	All code used to perform analysis can be found at: https://github.com/signeisacson/KIPF. Most of the analysis was performed in R ver. 4.3.2 with following packages: seqpac ver. 1.2.0, ggplot2 ver. 3.5.0, DESeq2 ver. 1.42.1, variancePartition ver. 1.32.5, stats ver. 4.3.2. Linear regression and normality check were performed with GraphPad Prism ver. 10.0.2.

For manuscripts utilizing custom algorithms or software that are central to the research but not yet described in published literature, software must be made available to editors and reviewers. We strongly encourage code deposition in a community repository (e.g. GitHub). See the Nature Portfolio guidelines for submitting code & software for further information.

Data

Policy information about availability of data

All manuscripts must include a data availability statement. This statement should provide the following information, where applicable:

- Accession codes, unique identifiers, or web links for publicly available datasets
- A description of any restrictions on data availability
- For clinical datasets or third party data, please ensure that the statement adheres to our policy

This study involves human participants and is covered by an ethical permit granted by the Swedish Ethics Board under number 2022-00244-01. Due to ethical considerations of the participants, raw sequencing data produced for this manuscript will not be publicly available. Normalised and filtered data are available in the supplementary material. Regarding safe sharing of raw data via extension of ethical permit, please contact anita.ost@liu.se.

Research involving human participants, their data, or biological material

Policy information about studies with human participants or human data. See also policy information about sex, gender (identity/presentation), and sexual orientation and race, ethnicity and racism.

Reporting on sex and gender	Our study investigates small RNA in sperm and its relation to clinical outcomes of in-vitro-fertilisation at the Center of Reproductive Medicine in Linkoping, Sweden. Eligible couples needed to be of male or female sex, as the population studied consisted of heterosexual couples. No questions of gender identity or sexual orientation were asked, and sex was assigned by clinical staff.
Reporting on race, ethnicity, or other socially relevant groupings	We have not collected any data regarding race, ethnicity or social groups in this study.
Population characteristics	In Sweden, couples get fertility treatment and care subsidised by tax funds if there are no biological babies previously in the relationship. To be eligible, females must be 25-40 years old and have a BMI < 30. Males 25-56 years of age. The couple must have had a stable relationship for 2 years and have tried conceiving for 1 year. For this study, specifically, males had to meet the following criteria: the presence of mature sperm in the ejaculate and proficiency in Swedish. Males were excluded if they had a known malignant disease, a history of radiation in the pelvic area, previous chemotherapy, or a known genetic anomaly. Importantly, only males with over 600,000 leftover sperm were sequenced due to a restriction in input RNA level.
Recruitment	Staff at the Centre of Reproductive Medicine recruited participants on the day of the oocyte pick-up. The recruitment was heavily dependent on the availability of responsible individuals on-site at the clinic. Eighty-four couples were assessed for eligibility; nine couples declined to participate, and three did not meet inclusion criteria. One risk of self-selection bias was that couples interested in research and academia participate, and are often academics themselves. Additionally, during certain weeks, men who did not wish to participate appeared in clusters, possibly due to heightened media attention around controversies of IVF in Sweden during the 1970s-1980s. Asked couples were not required to state reasons for declining, and no review has been conducted of the reasons behind our recruitment rate. The clinical setting may lead to couples to withhold reasons for their decline, afraid that it will affect their care, for example, undisclosed smokers, fearful of the distribution of genetic material.
Ethics oversight	Ethical permit was granted by Swedish Ethics Board under number 2022-00244-01.

Note that full information on the approval of the study protocol must also be provided in the manuscript.

Field-specific reporting

Please select the one below that is the best fit for your research. If you are not sure, read the appropriate sections before making your selection.

☒ Life sciences ☐ Behavioural & social sciences ☐ Ecological, evolutionary & environmental sciences

For a reference copy of the document with all sections, see nature.com/documents/nr-reporting-summary-flat.pdf

Life sciences study design

All studies must disclose on these points even when the disclosure is negative.

Sample size	72 couples were recruited. 65 treatments had a successful fertilisation. Sample size was evaluated based off prior literature in sperm-borne sRNA in humans, where sample size of n=54-87 (Hamilton et al. 2022, Hua et al. 2019) was capable of showing significant differences in sperm sRNA in a human population before.
Data exclusions	2 couples were excluded as no oocytes were retrieved from their treatment. This exclusion was performed post sample collection, as status of oocyte collection was unknown at time of sperm sample collection. No other data exclusion was performed. Samples were grouped differently for certain analysis. For each population studied per analysis, please see methods section of paper.
Replication	Laboratory methods are well described. All source code used for analysis is available to reproduce bioinformatic findings from this manuscript. Results were compared against other studies (Supplementary Table 10), but little reproducibility between our finding and others were found. This may be due to technical limitations, as sequencing depth was much greater in current manuscript than in other data publically available

	as of now.
Randomization	No randomization occured. In statistical model, covariate for technical batch effect for sequencing was accounted for. Covariates such as age and BMI was investigated for influence of data (Supplementary Figure S7).
Blinding	No blinding was performed, as no group allocation was performed during sample collection

Reporting for specific materials, systems and methods

We require information from authors about some types of materials, experimental systems and methods used in many studies. Here, indicate whether each material, system or method listed is relevant to your study. If you are not sure if a list item applies to your research, read the appropriate section before selecting a response.

Materials & experimental systems

n/a	Involved in the study
☒	☐ Antibodies
☒	☐ Eukaryotic cell lines
☒	☐ Palaeontology and archaeology
☒	☐ Animals and other organisms
☐	☒ Clinical data
☒	☐ Dual use research of concern
☒	☐ Plants

Methods

n/a	Involved in the study
☒	☐ ChIP-seq
☒	☐ Flow cytometry
☒	☐ MRI-based neuroimaging

Clinical data

Policy information about clinical studies

All manuscripts should comply with the ICMJEguidelines for publication of clinical research and a completedCONSORT checklist must be included with all submissions.

Clinical trial registration	this is not an intervention trial
Study protocol	this is not an intervention trial
Data collection	this is not an intervention trial
Outcomes	this is not an intervention trial

Plants

Seed stocks	-
Novel plant genotypes	-
Authentication	-
